# Risks and benefits of stress ulcer prophylaxis in adult neurocritical care patients: a systematic review and meta-analysis of randomized controlled trials

**DOI:** 10.1186/s13054-015-1107-2

**Published:** 2015-11-17

**Authors:** Bolin Liu, Shujuan Liu, Anan Yin, Javed Siddiqi

**Affiliations:** Division of Neurosurgery, Arrowhead Regional Medical Center, 400 North Pepper Avenue, Colton, CA 92324 USA; Department of Obstetrics and Gynecology, Xijing Hospital, Fourth Military Medical University, Xi’an, People’s Republic of China; Department of Neurosurgery, Xijing Institute of Clinical Neuroscience, Xijing Hospital, Fourth Military Medical University, Xi’an, People’s Republic of China

## Abstract

**Introduction:**

Neurocritical care patients are at high risk for stress-related upper gastrointestinal (UGI) bleeding. The aim of this meta-analysis was to evaluate the risks and benefits of stress ulcer prophylaxis (SUP) in this patient group.

**Methods:**

A systematic search of major electronic literature databases was conducted. Eligible studies were randomized controlled trials (RCTs) in which researchers compared the effects of SUP (with proton pump inhibitors or histamine 2 receptor antagonists) with placebo or no prophylaxis in neurocritical care patients. The primary outcome was UGI bleeding, and secondary outcomes were all-cause mortality and nosocomial pneumonia. Study heterogeneity was sought and quantified. The results were reported as risk ratios/relative risks (RRs) with 95 % confidence intervals (CIs).

**Results:**

We included 8 RCTs comprising an aggregate of 829 neurocritical care patients. Among these trials, one study conducted in a non–intensive care unit setting that did not meet our inclusion criteria was ultimately included based on further evaluation. All studies were judged as having a high or unclear risk of bias. SUP was more effective than placebo or no prophylaxis at reducing UGI bleeding (random effects: RR 0.31; 95 % CI 0.20–0.47; *P* < 0.00001; *I*^2^ = 45 %) and all-cause mortality (fixed effects: RR 0.70; 95 % CI 0.50–0.98; *P* = 0.04; *I*^2^ = 0 %). There was no difference between SUP and placebo or no prophylaxis regarding nosocomial pneumonia (random effects: RR 1.14; 95 % CI 0.67–1.94; *P* = 0.62; *I*^2^ = 42 %). The slight asymmetry of the funnel plots raised the concern of small trial bias, and apparent heterogeneity existed in participants, interventions, control treatments, and outcome measures.

**Conclusions:**

In neurocritical care patients, SUP seems to be more effective than placebo or no prophylaxis in preventing UGI bleeding and reducing all-cause mortality while not increasing the risk of nosocomial pneumonia. The robustness of this conclusion is limited by a lack of trials with a low risk of bias, sparse data, heterogeneity among trials, and a concern regarding small trial bias.

**Trial registration:**

International Prospective Register of Systematic Reviews (PROSPERO) identifier: CRD42015015802. Date of registration: 6 Jan 2015.

**Electronic supplementary material:**

The online version of this article (doi:10.1186/s13054-015-1107-2) contains supplementary material, which is available to authorized users.

## Introduction

Stress ulcer and stress-related upper gastrointestinal (UGI) bleeding is common in critically ill patients. The reported incidence of overt UGI bleeding ranges from 0.1 % to 4 % among all intensive care unit (ICU) patients and was up to 15 % among patients who received no prophylaxis, which was associated with worsened outcomes [1, 2].

Both the American Society of Health-System Pharmacists (ASHP) and the Surviving Sepsis Campaign guidelines recommend stress ulcer prophylaxis (SUP) with either histamine 2 receptor antagonists (H2RAs) or proton pump inhibitors (PPIs) as standard care in patients with a high risk of stress-related UGI bleeding [3, 4]. However, the rationale and level of evidence for this recommendation have been questioned by the findings of some recent randomized controlled trials (RCTs) and meta-analyses [1, 5–7]. Even though two recent meta-analyses showed that PPIs were more effective than H2RAs in preventing UGI bleeding in critically ill patients without increasing the risk of nosocomial pneumonia [8, 9], one meta-analysis and trial sequential analysis comparing the use of SUP versus no prophylaxis or placebo found that the quality and quantity of evidence for the use of SUP in adult ICU patients is low and that there is no firm evidence for benefit or harm of SUP [5].

Neurocritical care patients, however, are a unique subgroup. Neurological injury is an acknowledged risk factor for UGI bleeding, and the ASHP guidelines also recommend SUP for ICU patients with either an inability to obey simple commands or a Glasgow Coma Scale (GCS) score ≤10 [3]. The risk is potentiated by other major risk factors, including mechanical ventilation, hypotension, and coagulopathy [1]. Neurological injury, combined with severe physiological stress and critical illness, has been shown to increase the morbidity and mortality associated with stress-related UGI bleeding in the setting of acute neurological diseases, including traumatic brain injury (TBI) [10–14], spontaneous intracerebral hemorrhage (ICH) [15, 16], ischemic stroke [17–19], spinal cord injury [20, 21], central nervous system (CNS) infections, and so forth [22, 23].

Therefore, the findings of a meta-analysis including heterogeneous critically ill patients may not necessarily apply, and uncertainty over whether routine SUP is indicated in neurocritical care patients exists among clinicians. New evidence from one RCT favors the prophylactic use of PPIs over H2RAs or placebo in critically ill neurosurgical patients with ICH [16], which has emerged after a report of the most recent meta-analysis on this topic in general ICU patients [5]. We performed a systematic review and meta-analysis to weigh the risks of SUP against the benefits to answer the following research question: Is SUP with PPIs or H2RAs in neurocritical care patients superior to placebo or no prophylaxis in terms of UGI bleeding, all-cause mortality, and nosocomial pneumonia?

## Methods

This systematic review was conducted mainly using the methodology recommended by the Cochrane Collaboration [24], despite some necessary adaptations customized to the topic, and the review was prepared according to the PRISMA statement [25]. The protocol is published in the International Prospective Register of Systematic Reviews (PROSPERO identifier: CRD42015015802).

### Eligibility criteria

#### Types of studies

RCTs were eligible for inclusion.

#### Population

The study population was adult patients (age ≥18 years, without an upper limit) who received critical care for at least one of the following conditions: TBI, subarachnoid hemorrhage, ICH, ischemic stroke, anoxic brain injury, spinal cord injury, CNS infections, or other acute neurological injuries.

#### Intervention

The intervention was patients receiving SUP with at least one intervention group of PPIs or H2RAs.

#### Control

The control group was patients receiving placebo or no prophylaxis.

### Outcome

UGI bleeding was the primary outcome of this meta-analysis. All-cause mortality and nosocomial pneumonia were secondary outcomes. The outcome measures were used as defined by the authors of the original trials.

We included studies regardless of language of publication and publication status. We excluded studies in animals, in pediatric patients, and those in which the authors reported only non-patient-centered outcomes such as gastric pH and gastric colonization. For RCTs involving mixed populations but not presenting separate data for neurocritical care patients, the pooled results were included only if >75 % of patients had a neurocritical care diagnosis [26].

### Search strategy

MEDLINE, Embase, the Cochrane Central Register of Controlled Trials, and the Cochrane Database of Systematic Reviews were searched from their inception date until the first week of February 2015. To identify RCTs involving neurocritical care patients, the Boolean operator “AND” was used to combine four search concepts: stress ulcer, SUP, neurocritical care, and clinical trials. These concepts were created using a combination of National Library of Medicine Medical Subject Headings terms and keywords, and they were combined using the Boolean operator “OR” (Additional file 1: Appendix).

A separate search was also performed to identify clinical trials involving general critical care patients with heterogeneous diagnostic categories in multisystem ICUs (Additional file 1: Appendix). Previously published meta-analyses were used to identify relevant articles [1, 5–9, 27]. We reviewed the retrieved studies to determine if separate results were reported specifically for neurocritical care patients.

### Study selection

The retrieved records were reviewed independently and in duplicate by two authors (BL and SL). By screening of the titles, abstracts, and keywords, studies that were obviously not relevant were excluded. The remaining studies were assessed in full text. Disagreements were resolved by consensus.

### Data extraction

Two authors (BL and SL) extracted the data independently and in duplicate using a data extraction form, which was developed according to the recommendations of the Cochrane Collaboration. The form was tested in several studies and well-customized to the topic of the present review. The extracted information included trial characteristics (title, author, year of publication, country, trial design, duration, publication status, funding), the characteristics of the trial participants (number, age, sex, diagnosis, Acute Physiology and Chronic Health Evaluation II score, GCS score, other clinical parameters, duration of follow-up, dropout rates, inclusion criteria, risk factors for UGI bleeding, type of nutrition), exclusion criteria, type of intervention and/or control (name, dosing, duration, route of administration, comparator), and outcomes (clinically important bleeding, overt bleeding, occult bleeding, pneumonia, mortality, ICU length of stay, adverse events). Attempts were made to contact all the primary authors of the publications for missing data elements, and more than half responded with useful additional information [13, 15, 16, 22, 28].

### Risk of bias assessment

Risk of bias was assessed independently and in duplicate by two authors (BL and AY), using the tool recommended by the Cochrane Collaboration, including domains of random sequence generation, allocation concealment, blinding, incomplete outcome data, selective outcome reporting, baseline imbalance, and other bias [24]. Disagreements were resolved by consensus. The overall risk of bias for an individual trial was categorized as “low” (if the risk of bias was low in all domains), “unclear” (if the risk of bias was unclear in at least one domain, with no high risk of bias domains), or “high” (if the risk of bias was high in at least one domain) [24].

### Statistical analysis

Statistical analysis was performed using RevMan 5.3 software. The risk ratio/relative risk (RR) of each outcome measure was calculated with 95 % confidence interval (CI). Statistical heterogeneity was assessed by the *I*^2^ statistic [24]. *I*^2^ values of approximately 25 %, 50 %, and 75 % represent low, moderate, and high heterogeneity, respectively [9]. Substantial heterogeneity was predefined as *P* < 0.10 with *I*^2^ > 50 %. We used a fixed effects model if the *I*^2^ statistic was 0; otherwise, we used a random effects model. A test of interaction (with a *P* value <0.05 considered significant) was performed for each of the subgroups to examine the difference in effect size between two subgroups. Publication bias was assessed by funnel plot asymmetry [29].

### Subgroup analyses

To address heterogeneity potentially influencing estimated intervention effects, several strategies were used. Extensive subgroup analyses were conducted, including four predefined subgroup analyses: (1) lower (low or unclear) versus higher (high) risk of bias trials (possible smaller in trials with lower risk of bias [30]); (2) adequate versus inadequate random sequence generation, allocation concealment, and blinding (possibly smaller in trials with adequate random sequence generation, allocation concealment, and blinding [30]); (3) use of PPIs versus H2RAs (possibly larger in trials using PPIs [8, 9]); and (4) placebo trials versus no prophylaxis trials (possibly larger in trials using no prophylaxis [31]). We also conducted three post hoc subgroup analyses: (1) presence of enteral nutrition versus no enteral nutrition (possible larger intervention effect and increased risk of nosocomial pneumonia in trials using enteral nutrition [7]), (2) patients with TBI versus patients with ICH (possible different intervention effect), and (3) trials conducted in Asian versus non-Asian countries (possibly larger in Asian countries [32]). A post hoc random effects model was preferred even if statistical heterogeneity was not significant when apparent clinical and/or methodological diversity was judged to exist.

## Results

**Fig. 1:**
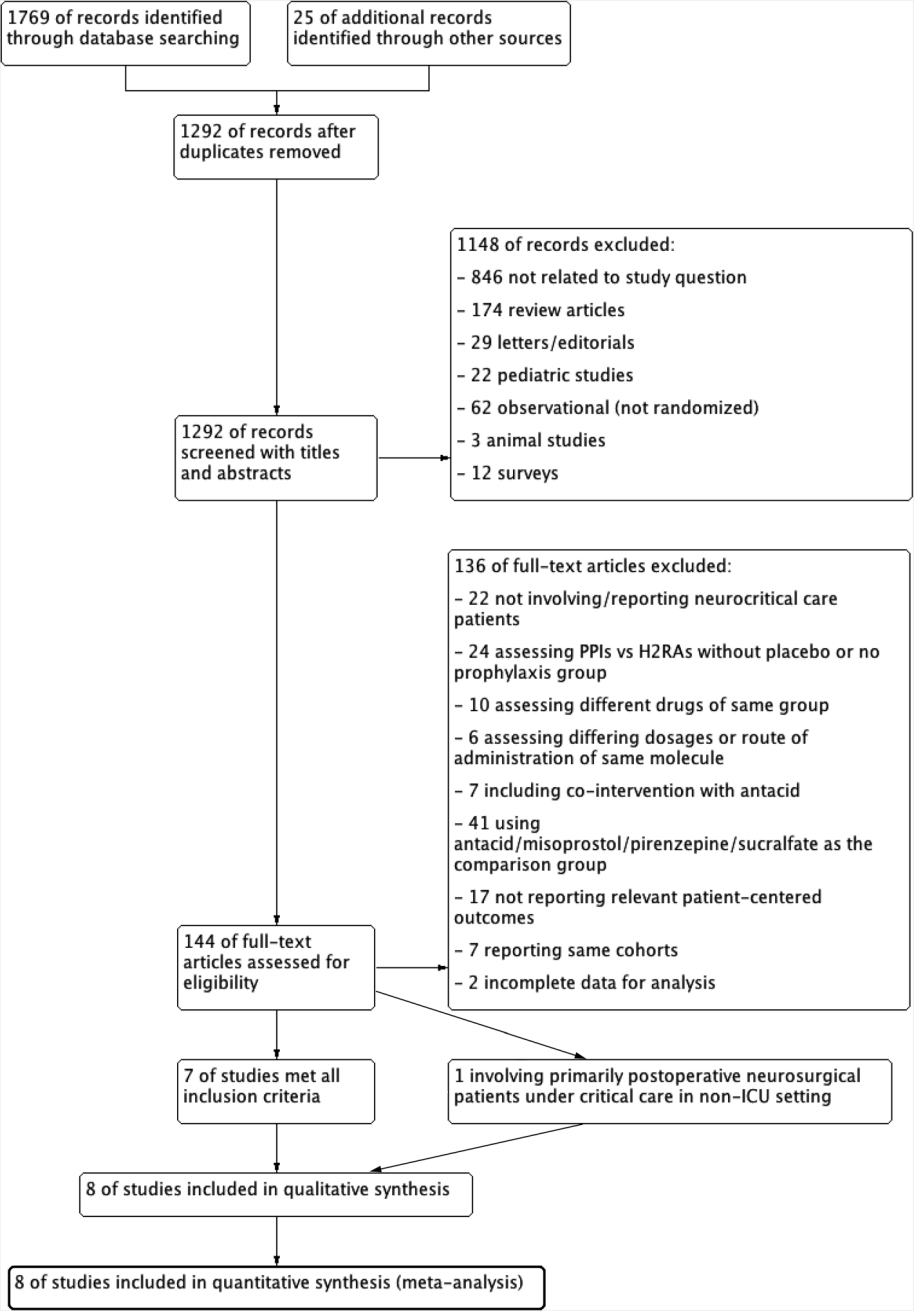
Study flow diagram. *H2RA* histamine 2 receptor antagonist, *ICU* intensive care unit, *PPI* proton pump inhibitor

The study selection process is summarized in Fig. 1. Eight studies involving an aggregate of 829 patients were included [11–16, 28]. The main reasons for exclusion were not involving and/or reporting neurocritical care patients and not including a placebo or no prophylaxis group. It is noteworthy that one study conducted in a non-ICU setting but consisting of patients who were critically ill with acute neurological injuries (median GCS 6, range 3–8) and underwent emergency neurosurgery and actually were under critical care perioperatively was also included [22]. Because this trial was a relatively large study with all included patients having a high risk of developing stress ulcers, and despite the appropriateness of excluding patients who underwent nonelective neurosurgery being debatable per se, the results were included in the main analysis. This is a protocol deviation, as we did include patients receiving perioperative critical care outside an ICU. A sensitivity analysis was done to validate the results by excluding the trial conducted in a non-ICU setting.

### Characteristics of included trials

The characteristics of the eight included trials are summarized in Table 1. The definition of primary outcome varied among the trials, with one reporting clinically important UGI bleeding [22], six reporting overt UGI bleeding [11–16], and one reporting seemingly overt UGI bleeding without a specified definition [28].

**Table 1:** Characteristics of the included studies

Study	Patients, *n*	Lost to follow-up, *n* (%)^a^	Setting/country	Trial duration (mo)	Diagnosis	EN	Inclusion criteria (population)	Exclusion criteria	Intervention	Comparator	Outcomes	UGI bleeding definition
Control: no prophylaxis												
Reusser et al., 1990 [11]	40	57/97 (59 %)	Single center/Switzerland	26	TBI, ICH	No	Severe acute traumatic or spontaneous hemorrhage intracranial lesion and neurosurgery and MV >48 h	Age <15 yr, GI surgery, PUD, SUP, UGI bleeding	Ranitidine 50 mg IV every 8/6 h titrated to maintain gastric pH ≥4	No prophylaxis	Overt UGI bleeding, mortality	Bright red bleeding via NG tube, melena, or decrease of blood hemoglobin level >2 g/dl within 24 h associated with a positive stool guaiac test or with gastric drainage of >100 ml of coffee-ground material
Control: placebo												
Burgess et al., 1995 [12]	34	0 (0 %)	Single center/United States	9	TBI	No	Severe head injury and GCS ≤10	PUD, GI injury, SUP, oral intake	Ranitidine 6.25 mg/h continuous IV for up to 72 h	Placebo	Overt UGI bleeding, mortality	Hematemesis, hematochezia, bright red blood per NG tube or coffee-ground NG tube aspirates, and a 5 % decrease from baseline in hematocrit occurring at least 8 h after study drug initiation
Chan et al., 1995 [22]	101	0 (0 %)	Single center/China	17	CVD, brain tumor, CNS infection, hydrocephalus	Yes	Nontraumatic cerebral disease and at least two risk factors for UGI bleeding^b^	UGI bleeding; chronic GI disease; PUD; concurrent heart, lung, kidney, hematological, and liver diseases	Ranitidine 50 mg IV every 6 h or 150 mg PO every 12 h when starting EN	Placebo	Clinically important UGI bleeding, nosocomial pneumonia	Gastroduodenal bleeding requiring blood transfusions and/or surgery for acute perforated ulcers, lesions confirmed either endoscopically or during abdominal surgery
Halloran et al., 1980 [13]	50	0 (0 %)	Single center/United States	20	TBI	Yes	Severe head injury and neurological deficits	Apnea and fixed dilated pupils and no motor response, PUD, pregnancy, GI injury, severe hepatic or renal disease	Cimetidine 300 mg IV every 4 h for up to 3 wk	Placebo	Overt UGI bleeding, mortality	Bright red blood or a 4+ positive stool guaiac test in the gastric aspirate for three consecutive 8-h periods (exclusive of first day after injury), excluding oropharyngeal source of bleeding
Liu et al., 2013 [16]	165	19/184 (10 %)	Single center/China	32	ICH	No	CT-proven ICH within 72 h of ictus and neurosurgery, NG tube in place, baseline gastric pH <4, negative GOBT, age >18 yr	AVM, PUD, facial trauma, anticoagulants, AKI, thrombocytopenia, died within 72 h after ictus	Omeprazole 40 mg IV every 12 h for up to 7 days, cimetidine 300 mg IV every 6 h for up to 7 days	Placebo	Overt UGI bleeding, mortality, nosocomial pneumonia	Hematemesis, aspiration of coffee-ground material from NG tube, or melena, proven by positive GOBT or FOBT, with or without hemodynamic instability resulting from gross bleeding that needed transfusion
Metz et al., 1993 [14]	167	0 (0 %)	Multicenter/United States	20	TBI	No	Severe head injury with 24 h of injury and GCS ≤10, NG tube in place, age >18 yr, expected ICU stay ≥72 h	GI bleeding, severe burns >20 %, AKI, PUD, thrombocytopenia, SUP	Ranitidine 6.25 mg/h continuous IV for up to 5 days	Placebo	Overt UGI bleeding, nosocomial pneumonia	• Gastroccult positive NG tube drainage and coffee-ground material for the previous 8 h • Minimum of 50 ml bright red blood per NG tube • Hematemesis in the last 8 h • Hemoccult positive stool • Melena • Hematochezia; with or without endoscopic or surgical confirmation of UGI source of bleeding
Misra et al., 2005 [15]	141	35/176 (20 %)	Single center/India	24	ICH	Yes	CT-proven ICH within 7 days of ictus	AVM, coagulopathy, hepatic or renal disease, PUD, anticoagulants	Ranitidine 50 mg IV every 8 h	Placebo	Overt UGI bleeding, mortality, nosocomial pneumonia	Gross blood, coffee-ground aspirate from NG tube, hematemesis or melena
Zhang et al., 2014 [28]	180	0 (0 %)	Single center/China	NA	ICH	Yes	CT-proven ICH within 72 h of ictus, age 30–75 yr	Traumatic or brain tumor-related hemorrhage, coagulopathy, PUD, mental disorder or dementia, concurrently included in other clinical trials	Esomeprazole 40 mg/day (*n* = 36) or lansoprazole 40 mg/day (*n* = 36) PO, ranitidine 150 mg/day (*n* = 36) or famotidine 40 mg/day (*n* = 36) PO	Placebo	Overt UGI bleeding	Clinical evidence of GI bleeding reported, but definition not specified (endoscopy used in all patients at approximately day 21 since SUP)

*AKI* acute kidney injury, *AVM* arteriovenous malformation, *CNS* central nervous system, *CT* computed tomography, *CVD* cerebrovascular disease, *EN* enteral nutrition, *FOBT* fecal occult blood test, *GCS* Glasgow Coma Scale, *GI* gastrointestinal, *GOBT* gastric occult blood test, *ICH* intracerebral hemorrhage, *IV* intravenous, *MV* mechanical ventilation, *NA* not available, *NG* nasogastric, *PO* per os, *PUD* peptic ulcer disease, *SUP* stress ulcer prophylaxis, *TBI* traumatic brain injury, *UGI* upper gastrointestinal

^a^Number and percentage of patients lost to follow-up and due to other reasons not included in the analysis for the primary outcome among all eligible patients

^b^Risk factors included preoperative coma (GCS <9), inappropriate secretion of antidiuretic hormone, major postoperative complications requiring reoperation, age ≥60 yr, and pyogenic CNS infection

### Participants

In 5 trials all eligible patients were included in the study analysis [12–14, 22, 28], and in 3 trials 111 (24.3 %) of 457 patients were excluded from the analysis due to loss to follow-up and other reasons, including enrollment error, missing data/procedures, lack of consent, bleeding not related to stress ulcer, and early death [11, 15, 16]. Of the 829 patients included in the analysis in all 8 trials, 288 from 4 trials had TBI [11–14], 440 had ICH [11, 15, 16, 28], and the remainder were emergency neurosurgical patients (69 had cerebrovascular disease not specified, 29 had brain tumors, 3 had CNS infections, and 10 had hydrocephalus) [22]. The mean ages of the patients ranged from 29.6 to 61.0 years, and the male-to-female ratio was 1.71:1 (523 vs. 306, respectively). GCS scores were reported in six trials, and the mean GCS score ranged from 5 to 9.8 [11, 12, 14–16, 22]. For each trial, patients’ baseline characteristics in all intervention and control groups were well-balanced. In four trials, patients received enteral nutrition [13, 15, 22, 28].

### Interventions and controls

Six trials used H2RA as an intervention [11–15, 22] (one trial used both H2RA and sucralfate as interventions, but the sucralfate group was not included in this meta-analysis), and two trials used more than one intervention (both PPI and H2RA) [16, 28]. The route of administration was intravenous in six trials [11–16], orally in one trial [28], and either orally or intravenously in one trial [22]. Regarding control treatment, placebo was used as a comparator in seven trials [12–16, 22, 28] and no prophylaxis was used in one trial [11].

### Risk of bias assessment

**Fig. 2:**
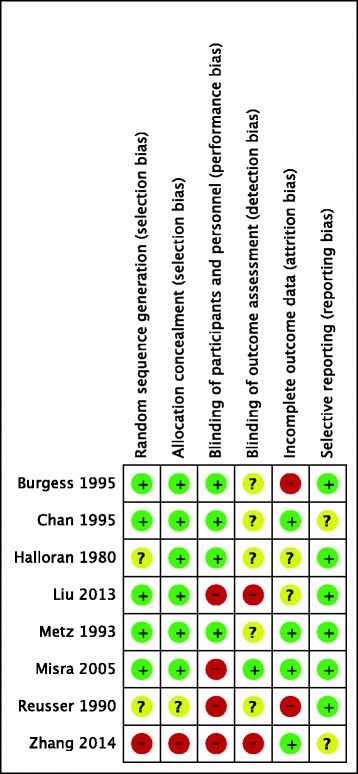
Risk of bias summary of review authors’ judgments about each risk of bias item for each included study. *Red* high risk, *green* low risk, *yellow* unclear

No trials were judged to have a low risk of bias in all domains. Three trials had unclear risk of bias [13, 14, 22], and the remaining five trials had a high risk of bias [11, 12, 15, 16, 28] (Fig. 2). It is noteworthy that our judgments regarding the risk of bias in some studies differed from previous systematic reviews [5]. The variation may result from different information identified in the study reports and different interpretation of the tool [33]. The statements supporting our judgment for each included study are provided in Additional file 2. The main reasons for high risk of bias were inadequate blinding or incomplete outcome data. No trials met the criteria of adequate random sequence generation, allocation concealment, and blinding. Therefore, one of the predefined subgroup analyses of trials with adequate versus inadequate random sequence generation, allocation concealment, and blinding could not be done.

### Outcome measures

#### UGI bleeding

**Fig. 3:**
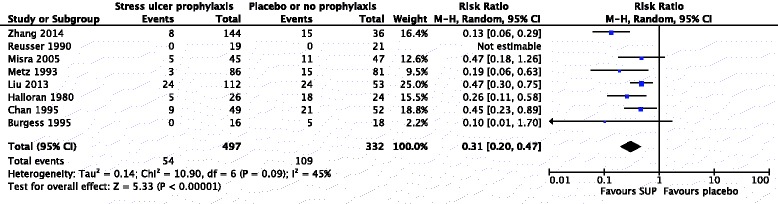
Forest plot of stress ulcer prophylaxis (SUP) and upper gastrointestinal bleeding. *CI* confidence interval, *M-H* Mantel–Haenszel method

UGI bleeding data were available in all eight trials [11–16, 22, 28]. The median duration of follow-up ranged from 2 to 20 days. SUP in neurocritical care patients resulted in a lower incidence of UGI bleeding than treatment with placebo or no prophylaxis (11 % vs. 33 %; random effects: RR 0.31; 95 % CI 0.20–0.47; *P* < 0.00001; *P* = 0.09 for heterogeneity; *I*^2^ = 45 %) (Fig. 3). Subgroup analyses showed no significant heterogeneity among the included trials regarding different interventions (*P* = 0.42 for interaction of PPIs vs. H2RAs) (Table 2) or different neurological pathologies (*P* = 0.52 for interaction of TBI vs. ICH) (Table 2). However, more complex neurological care conditions than our subgroup analyses could cover were observed across the trials in the areas of disease states and brain injury severity, which may influence the pathophysiology of stress ulcer and the benefits of SUP and thus have compromised the our results. Visual inspection of a funnel plot showed slight asymmetry at the bottom of the funnel plot (Additional file 3: Figure S1).

**Table 2:** Subgroup analyses for outcomes

Comparison	UGI bleeding					Mortality					Nosocomial pneumonia				
	Studies (*n*)	Patients (*n*)	RR (95 % CI)	*P* value^a^	*I* ^2^ value^b^	Studies (*n*)	Patients (*n*)	RR (95 % CI)	*P* value^a^	*I* ^2^ value^b^	Studies (*n*)	Patients (*n*)	RR (95 % CI)	*P* value^a^	*I* ^2^ value^b^
Risk of bias				0.92	0 %				0.32	0 %				0.82	0 %
Higher (high)	5	527	0.30 (0.16–0.56)			4	347	0.68 (0.48–0.95)			2	257	0.98 (0.29–3.31)		
Lower (low/unclear)	3	302	0.32 (0.15–0.66)			1	34	3.35 (0.15–76.93)			2	264	1.16 (0.51–2.63)		
Intervention				0.42	0 %				0.68	0 %				0.46	0 %
PPIs	2	219	0.20 (0.06–0.68)			1	111	0.78 (0.46–1.32)			1	111	1.60 (0.73–3.51)		
H2RAs	8	699	0.35 (0.22–0.53)			5	323	0.68 (0.47–0.98)			4	463	1.12 (0.66–1.91)		
Control				NA	NA				0.57	0 %				NA	NA
Placebo	7	789	0.31 (0.20–0.47)			4	341	0.68 (0.47–0.97)			4	521	NA		
No prophylaxis	1	40	Not estimable			1	40	0.92 (0.33–2.53)			0	0	NA		
Enteral nutrition				0.86	0 %				0.31	2.9 %				0.96	0 %
Yes	4	423	0.29 (0.16–0.53)			2	142	0.55 (0.31–1.00)			2	193	1.03 (0.26–4.01)		
No	4	406	0.32 (0.14–0.74)			3	239	0.80 (0.53–1.21)			2	328	1.07 (0.53–2.15)		
Diagnosis				0.52	0 %				0.36	0 %				0.71	0 %
TBI	4	291	0.22 (0.11–0.43)			3	124	0.88 (0.49–1.57)			1	163	0.75 (0.38–1.51)		
ICH	3	437	0.31 (0.14–0.72)			2	257	0.63 (0.41–0.95)			2	257	0.98 (0.29–3.31)		
Study location				0.31	1.6 %				0.36	0 %				0.17	46.0 %
Asia	4	538	0.35 (0.20–0.62)			2	257	0.63 (0.41–0.95)			3	358	1.40 (0.80–2.47)		
Non-Asian	4	291	0.22 (0.11–0.43)			3	124	0.88 (0.49–1.57)			1	163	0.75 (0.38–1.51)		

*CI* confidence interval; *H2RA* histamine 2 receptor antagonist, *ICH* intracerebral hemorrhage, *NA* not applicable, *PPI* proton pump inhibitor, *RR* risk ratios, *TBI* traumatic brain injury, *UGI* upper gastrointestinal

^a^ *P* values for interaction between groups

^b^ *I* ^2^ values for heterogeneity between groups

#### All-cause mortality

**Fig. 4:**
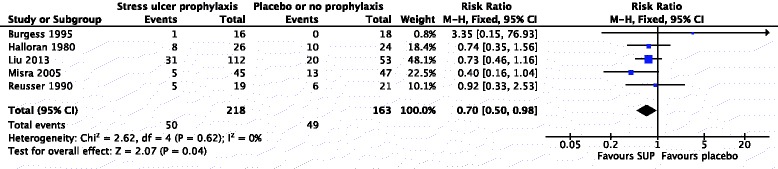
Forest plot of stress ulcer prophylaxis (SUP) and all-cause mortality. *CI* confidence interval, *M-H* Mantel–Haenszel method

Mortality data were available in 5 trials involving 381 patients [11–13, 15, 16]. The duration of follow-up ranged from 3 to 30 days, and the median time to the final follow-up assessment for mortality was 20 days. There was a statistically significant decrease in all-cause mortality of patients receiving SUP versus placebo or no prophylaxis (23 % vs. 30 %; fixed effects: RR 0.70; 95 % CI 0.50–0.98; *P* = 0.04; *P* = 0.62 for heterogeneity; *I*^2^ = 0 %) (Fig. 4). A post hoc random effects model considering possible clinical heterogeneity showed results consistent with the main analysis (random effects: RR 0.71; 95 % CI 0.51–0.99; *P* = 0.04), as well as all the subgroup analyses regarding all-cause mortality (data not shown). Visual inspection of a funnel plot showed slight asymmetry (Additional file 4: Figure S2).

#### Nosocomial pneumonia

**Fig. 5:**
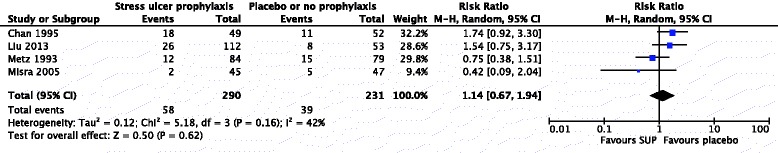
Forest plot of stress ulcer prophylaxis (SUP) and nosocomial pneumonia. *CI* confidence interval, *M-H* Mantel–Haenszel method

In 4 trials involving 521 patients, researchers reported the incidence of nosocomial pneumonia [14–16, 22]. However, only two of the four trials included explicit definitions of this outcome (they were still different between the two) [14, 22]. The proportion of patients receiving SUP who developed nosocomial pneumonia varied between studies, ranging from 4 % to 37 %. The incidence of nosocomial pneumonia was no greater among patients receiving SUP than among those receiving placebo or no prophylaxis (20 % vs. 17 %; random effects: RR 1.14; 95 % CI 0.67–1.94; *P* = 0.62; *P* = 0.16 for heterogeneity; *I*^2^ = 42 %) (Fig. 5). Either the lack or the variation in definitions of outcome measures, different disease severity, and complex neurological care conditions may account for the possible heterogeneity and thus may have compromised the results. Visual impression revealed a similar slight asymmetry in a funnel plot (Additional file 5: Figure S3).

#### Subgroup and sensitivity analyses

We found no significant subgroup heterogeneity regarding UGI bleeding, all-cause mortality, or nosocomial pneumonia when we compared trials with higher (high) versus lower (low/unclear) risk of bias, intervention drugs (PPIs vs. H2RAs), comparator types (placebo vs. no prophylaxis), presence of nutrition versus no enteral nutrition, diagnosis (TBI vs. ICH), and study location (Asia vs. non-Asian). The results, including *P* values for interactions, are summarized in Table 2.

In sensitivity analysis, exclusion of the trial conducted in a non-ICU setting but involving critically ill patients who underwent emergency neurosurgeries had little impact on the results [22]. Specifically, UGI bleeding (random effects: RR 0.28; 95 % CI 0.16–0.47; *P* < 0.00001; *I*^2^ = 50 %) was significantly reduced and risk of nosocomial pneumonia (random effects: RR 0.94; 95 % CI 0.49–1.78; *P* = 0.84; *I*^2^ = 37 %) was not increased, consistent with the main analysis.

## Discussion

In this meta-analysis, SUP with either H2RAs or PPIs in adult neurocritical care patients was more effective than placebo or no prophylaxis in reducing UGI bleeding and all-cause mortality without increasing the risk of nosocomial pneumonia. However, the main results were limited by the lack of trials with low risk of bias, sparse data, heterogeneity among trials, and concern regarding small trial bias.

### UGI bleeding

The mechanisms responsible for stress ulcerations are complex, including multiple factors affecting the imbalance between mucosal protection and gastric acid production. Compared with general ICU patients, critically ill neurosurgical and neurological patients are more vulnerable to stress-related UGI bleeding, as they have increased acid secretion caused by stress-triggered vagal stimulation of the stomach through CNS pathways [18, 23, 34] and impaired mucosal protection resulting from compromised mucosal microcirculation [35]. In addition, the presence of elevated intracranial pressure (ICP) can be found in any mechanism of cerebral injury, which is a well-known contributing factor in stress ulcer formation [15, 16, 23, 36]. These risk factors related to neurological injury could be potentiated by other risk factors, including mechanical ventilation, hypotension, and coagulopathy, which are common in neurocritical care patients as well [1, 23]. Indeed, several studies have shown that neurocritical care patients have increased morbidity and mortality associated with stress-related UGI bleeding [10–23]. In addition, because this increased risk of UGI bleeding is a consequence of elevated ICP, cerebral ischemia, sympathetic hyperactivity, and the inflammatory response that exist in almost all neurocritical care patients [23], it is unlikely that one specific type of neurological injury (such as TBI vs. ICH) predisposes patients to a higher risk than another. Therefore, neurocritical care patients in general warrant special attention to this issue in practice.

The present meta-analysis of UGI bleeding showed a benefit of SUP over placebo or no prophylaxis. However, this apparent benefit of SUP should be conservatively interpreted because (1) the pooled analysis may be influenced by the relatively lower quality of the included trials, as no trial in this meta-analysis was judged to be of low risk of bias in all domains and no single trial had adequate random sequence generation, allocation concealment, and blinding; (2) the slight asymmetry of the funnel plot raised the concern of small trial bias; (3) apparent heterogeneity in participants, interventions, control treatments, and outcome measures did exist among the included studies; and (4) the size of this meta-analysis is small, and thus its power is limited. Taken together, the effect size of benefit with SUP may have been biased toward a larger effect.

### All-cause mortality

Stress-related UGI bleeding has been shown to be a strong predictor of mortality in critically ill patients, which is associated with a mortality rate of 50–77 % and is as much as four times higher than that of patients without UGI bleeding [37]. Our pooled analysis of all-cause mortality showed a benefit of SUP in neurocritical care patients, and the robustness of the findings was confirmed by the sensitivity analyses. Despite these observations, however, it is still elusive whether stress-related UGI bleeding is a contributing cause of mortality or simply a marker of disease severity. Because the median duration of follow-up for mortality was only 20 days, the long-term outcome of patients who may or may not have benefited from SUP is unknown. Additionally, a genuine benefit of SUP on mortality might be questioned concerning the similar bias that existed in the analysis of UGI bleeding.

### Nosocomial pneumonia

One of the proposed complications of SUP is an increased vulnerability to nosocomial pneumonia. However, we could not find any benefit or harm of SUP on the risk of nosocomial pneumonia. The overall higher risk of bias of the trials and sparse data warrant cautious interpretation of the results. Though not significant, as shown by subgroup analyses, between-trial heterogeneity was observed, which is possibly due to (1) clinical variability in participants, interventions, and control treatments; (2) unclear or inconsistent definitions of nosocomial pneumonia [14–16, 22]; and (3) inherent bias associated with adverse effect outcomes in RCTs, as they are not always foreseeable and may not have been adequately addressed in the original studies [24].

### Protocol deviations

There are a few protocol deviations in this meta-analysis compared with the original published protocol in PROSPERO. We did not perform Egger’s test and meta-regression, because there were fewer than ten trials included and in that case the statistical power of these tests is very confined [24]. We included a trial conducted in a non-ICU setting with perioperative critically ill patients [22], and to explore the impact of this protocol deviation we performed a sensitivity analysis by excluding this trial. Although the intention is that a meta-analysis should adhere to the published protocol, changes in protocol are sometimes necessary to adapt to unanticipated circumstances, such as problems with participant recruitment, data collection, and unexpected event rates.

### Relationship to other reviews

Recent systematic reviews with meta-analyses indicated a benefit of PPIs over H2RAs in reducing the risk of UGI bleeding without affecting the risk of nosocomial pneumonia, death, or ICU length of stay for general critical care patients [8, 9]. However, the authors of these reviews have overlooked one important question: whether SUP with PPIs or H2RA has any benefit over placebo or no prophylaxis. There is a possibility that even though PPIs are better than H2RAs, neither may be better than placebo or no prophylaxis. In that case, the conclusions of the superior effects of PPIs may be of limited or no clinical significance.

Indeed, the authors of one recent systemic review using meta-analysis and trial sequential analysis questioned this issue by assessing the efficacy and safety of SUP versus placebo or no prophylaxis in general critical care patients [5]. The results revealed that SUP with PPIs or H2RAs was not statistically significantly different from placebo or no prophylaxis in terms of mortality, GI bleeding, and pneumonia. Yet, our results support the effectiveness and safety of SUP with PPIs or H2RAs in reducing the morbidity and mortality of stress-related UGI bleeding in neurocritical care patients. The difference could be attributed to three factors. First and foremost, the trial eligibility criteria varied significantly between the two reviews. Krag et al.’s [5] review included critically ill patients from medical, surgical, and mixed ICUs with various primary diagnoses and different disease states and severity. In contrast, our review included only neurocritical care patients with primary pathologies, all of which were related to CNS and comparable risks, and these patients were in general at greater risk for stress-related UGI bleeding and associated complications, as discussed above. Second, owing to the update of RCTs on SUP in critical ill patients, we included three recently published trial results [15, 16, 28] not included in Krag et al.’s review [5]. In addition, we included a trial conducted in a non-ICU setting that consisted of critically ill patients with acute and severe neurological injuries who underwent emergency neurosurgery and were under critical care perioperatively. This trial was excluded from Krag et al.’s review simply because it was not in an ICU setting; however, we decided to include it based on the evaluation of the characteristics of the patients and the actual level of care they received. Third, the statistical methods used were different. We did not perform a trial sequential analysis to challenge the meta-analysis, partially because an anticipated RR reduction of 20 % for intervention effect with an event proportion of 21 % in the control arm is somewhat too rigorous. However, the lack of a trial sequential analysis may limit the robustness of our results.

### Strengths and limitations of the review

The robustness of our findings is supported by general compliance with the recommendations of the Cochrane Collaboration for intervention reviews for RCTs, despite some necessary adaptations customized to the present topic.

However, our meta-analysis also has many limitations, such as the clinical and methodological heterogeneity, protocol deviations, sparse data, and concerns regarding small trial bias. Thus, the generalizability of the results may be compromised. In addition, our findings should not necessarily be applied to patients undergoing elective neurosurgical procedures, because these patients were not included in our analysis.

### Implications for future practice and research

The meta-analysis encouraged the use of SUP in neurocritical care patients by providing evidence that SUP with PPIs or H2RAs yields a reduction in stress-related UGI bleeding and all-cause mortality and does not increase the risk of nosocomial pneumonia compared with placebo or no prophylaxis. Recommendations in current guidelines that advocate SUP for patients with high risk of stress-related UGI bleeding [3, 4] are supported by this meta-analysis. However, there are no clear recommendations on the monitoring and discontinuation of SUP in critical ill patients to date, and this needs to be addressed in future investigations.

The overall high or unclear risk of bias of the trials and sparse data in this meta-analysis highlight the lack of firm evidence for the benefits of SUP in neurocritical care patients. Larger, well-designed RCTs with low risk of bias are thus warranted for the safety and effective care of patients. In addition, among all neurocritical care patients, almost all RCTs have been targeted at patients with TBI and ICH; patients with other acute neurological conditions, such as ischemic stroke, spinal cord injury, and aneurysmal subarachnoid hemorrhage, should be taken into consideration.

## Conclusions

This meta-analysis provides a comprehensive summary of available trial information for clinicians and guideline developers. Our results suggest that SUP, compared with placebo or no prophylaxis, may significantly lower the risk of UGI bleeding and all-cause mortality in neurocritical care patients without influencing the risk of nosocomial pneumonia. The robustness of this conclusion is limited by the lack of trials with low risk of bias, sparse data, heterogeneity among trials, and concern regarding small trial bias. Cost-effectiveness analysis and larger, well-designed RCTs are warranted among neurocritical care patients to allow firm conclusions to be drawn about the magnitude of the beneficial effect and its clinical relevance.

## Key messages

- Stress ulcer prophylaxis with proton pump inhibitors or histamine 2 receptor antagonists seems to be more effective than placebo or no prophylaxis in reducing the morbidity and mortality of stress-related upper gastrointestinal bleeding in neurocritical care patients.
- Stress ulcer prophylaxis seems to reduce all-cause mortality while not increasing the risk of nosocomial pneumonia in neurocritical care patients.
- The robustness of these conclusions is limited by the lack of trials with low risk of bias, sparse data, heterogeneity among trials, and concern regarding small trial bias.

## Additional files

Additional file 1:
**Appendix.** (DOCX 106 kb) Additional file 2:
**Risk of bias table.** (DOCX 109 kb) Additional file 3: Figure S1. Funnel plot: UGI bleeding. (EPS 81 kb) Additional file 4: Figure S2. Funnel plot: all-cause mortality. (EPS 81 kb) Additional file 5: Figure S3. Funnel plot: nosocomial pneumonia. (EPS 84 kb)

## Abbreviations

AKI: acute kidney injury
ASHP: American Society of Health-System Pharmacists
AVM: arteriovenous malfunction
CI: confidence interval
CNS: central nervous system
CT: computed tomography
CVD: cardiovascular disease
EN: enteral nutrition
FOBT: fecal occult blood test
GCS: Glasgow Coma Scale
GI: gastrointestinal
GOBT: gastric occult blood test
H2RA: histamine 2 receptor antagonist
ICH: intracerebral hemorrhage
ICP: intracranial pressure
ICU: intensive care unit
IV: intravenous
MV: mechanical ventilation
NA: not available
NG: nasogastric
PO: per os
PPI: proton pump inhibitor
PROSPERO: International Prospective Register of Systematic Reviews
PUD: peptic ulcer disease
RCT: randomized controlled trial
RR: risk ratio/relative risk
SUP: stress ulcer prophylaxis
TBI: traumatic brain injury
UGI: upper gastrointestinal
