# Supplementary material for: Risks and benefits of stress ulcer prophylaxis in adult neurocritical care patients: a systematic review and meta-analysis of randomized controlled trials
Source: Crit Care. 2015 Nov 17;19:409. doi: 10.1186/s13054-015-1107-2 (PMC4650140; doi:10.1186/s13054-015-1107-2)
Supplement: Additional file 1: — Appendix. (DOCX 106 kb) [file 13054_2015_1107_MOESM1_ESM.docx]

**Appendix - Search strategy**

**PICO question**

Population: critically ill OR icu OR intensive care unit OR intensive care

Intervention: stress ulcer prophylaxis OR sup OR ppi OR proton pump inhibitor OR pantoprazole OR omeprazole OR rabeprazole OR dexlansoprazole OR esomeprazole OR lansoprazole OR h2ra OR nizatidine OR famotidine OR cimetidine OR ranitidine OR histamine h2 antagonists

Comparator: control OR placebo

Outcomes: mortality OR death OR gi bleeding OR gastrointestinal bleeding OR pneumonia OR vap OR morbidity

I. MEDLINE search strategy used to identify randomized controlled trials assessing glycemic control strategies in neurocritical care patients (the EMBASE and COCHRANE search involved minor variations)

1. peptic ulcer/ (82421)
2. gastrointestinal hemorrhage/ (47445)
3. injury/ AND gastric mucosa/ (3686)
4. (ulcer* OR lesion*).mp. [mp=protocol supplementary concept, rare disease supplementary concept, title, original title, abstract, name of substance word, subject heading word, unique identifier] (850610)
5. stress ulcer/ (5699)
6. (bleed* OR re-bleed* OR rebleed* OR hemorrhage OR hematochezia OR hematemese OR melena).mp. [mp=protocol supplementary concept, rare disease supplementary concept, title, original title, abstract, name of substance word, subject heading word, unique identifier] (412569)
7. (stomach OR gastri* OR gastropathy OR epigastr* OR gastro-duodenal OR gastroduodenal OR oesophag* OR esphag* OR "upper gi" OR ugi OR "upper gastrointestinal").mp. [mp=protocol supplementary concept, rare disease supplementary concept, title, original title, abstract, name of substance word, subject heading word, unique identifier] (518616)
8. gastritis/ (25586)
9. OR/1-8 (1595935)
10. proton pump inhibitor/ (18808)
11. omeprazole/ (10573)
12. esomeprazole/ (1050)
13. lansoprazole / (2460)
14. dexlansoprazole/ (68)
15. pantoprazole/ (1492)
16. rabeprazole/ (1076)
17. histamine h2 antagonists/ (19704)
18. cimetidine/ (12248)
19. famotidine/ (2029)
20. nizatidine/ (399)
21. ranitidine/ (6670)
22. roxatidine/ (179)
23. (ppi OR h2ra OR roxatidine OR histamine 2 receptor antagonists).mp. [mp=protocol supplementary concept, rare disease supplementary concept, title, original title, abstract, name of substance word, subject heading word, unique identifier] (15520)
24. OR/10-23 (50013)
25. brain injuries/ (69756)
26. brain edema/ (22112)
27. craniocerebral trauma/ (125090)
28. cerebrovascular trauma/ (7943)
29. subarachnoid hemorrhage/ (23794)
30. cerebral hemorrhage/ (47681)
31. intracranial hemorrhage/ (65386)
32. brain ischemia/ (95130)
33. stroke/ (223787)
34. cerebral infarction/ (231030)
35. intracranial embolism/ (12714)
36. intracranial thrombosis/ (16107)
37. spinal cord injuries/ (44620)
38. heart arrest/ (48429)
39. hypoxia, brain/ (20875)
40. anoxia/ (61966)
41. (neurocritical* OR neuro-critical* OR neurolog* OR neurosurg* neurological intensive care OR neurologic intensive care OR traumatic brain injury OR brain trauma OR diffuse axonal injury OR subdural hematoma OR epidural hematoma OR intracerebral hemorrhage OR cerebrovascular accident OR cardiac arrest).mp. [mp=protocol supplementary concept, rare disease supplementary concept, title, original title, abstract, name of substance word, subject heading word, unique identifier] (1190767)
42. OR/25-41 (1190767)
43. randomized clinical trial/ (474963)
44. random allocation/ (83845)
45. clinical trial/ (1014634)
46. controlled clinical trial/ (195819)
47. single-blind method/ (34138)
48. double-blind method/ (129101)
49. placebo/ (178207)
50. (trial* OR random*).mp. [mp=protocol supplementary concept, rare disease supplementary concept, title, original title, abstract, name of substance word, subject heading word, unique identifier] (1605262)
51. OR/43-50 (1722995)
52. 9 AND 24 AND 42 AND 51 (92)

II. MEDLINE search strategy used to identify randomized contolled trials assessing glycemic control strategies in critically ill patients (same strategy used in reference 51; EMBASE and COCHRANE search involved minor variations).

1. peptic ulcer/ (82421)
2. gastrointestinal hemorrhage/ (47445)
3. injury/ AND gastric mucosa/ (3686)
4. (ulcer* OR lesion*).mp. [mp=protocol supplementary concept, rare disease supplementary concept, title, original title, abstract, name of substance word, subject heading word, unique identifier] (850610)
5. stress ulcer/ (5699)
6. (bleed* OR re-bleed* OR rebleed* OR hemorrhage OR hematochezia OR hematemese OR melena).mp. [mp=protocol supplementary concept, rare disease supplementary concept, title, original title, abstract, name of substance word, subject heading word, unique identifier] (412569)
7. (stomach OR gastri* OR gastropathy OR epigastr* OR gastro-duodenal OR gastroduodenal OR oesophag* OR esphag* OR "upper gi" OR ugi OR "upper gastrointestinal").mp. [mp=protocol supplementary concept, rare disease supplementary concept, title, original title, abstract, name of substance word, subject heading word, unique identifier] (518616)
8. gastritis/ (25586)
9. OR/1-8 (1595935)
10. proton pump inhibitor/ (18808)
11. omeprazole/ (10573)
12. esomeprazole/ (1050)
13. lansoprazole/ (2460)
14. dexlansoprazole/ (68)
15. pantoprazole/ (1492)
16. rabeprazole/ (1076)
17. histamine h2 antagonists/ (19704)
18. cimetidine/ (12248)
19. famotidine/ (2029)
20. nizatidine/ (399)
21. ranitidine/ (6670)
22. roxatidine/ (179)
23. (ppi OR h2ra OR roxatidine OR histamine 2 receptor antagonists).mp. [mp=protocol supplementary concept, rare disease supplementary concept, title, original title, abstract, name of substance word, subject heading word, unique identifier] (15520)
24. OR/10-23 (50013)
25. intensive care unit/ (116450)
26. intensive care/ (175928)
27. critical care/ (149742)
28. critical illness/ (30875)
29. coronary care unit/ (12455)
30. postoperative care/ (120035)
31. (icu OR coronary care OR recovery room OR par OR burn unit OR critically ill OR cardiac care).mp. [mp=protocol supplementary concept, rare disease supplementary concept, title, original title, abstract, name of substance word, subject heading word, unique identifier] (229416)
32. OR/25-31 (497429)
33. randomized clinical trial/ (474963)
34. random allocation/ (83845)
35. clinical trial/ (1014634)
36. controlled clinical trial/ (195819)
37. single-blind method/ (34138)
38. double-blind method/ (129101)
39. placebo/ (178207)
40. (trial* OR random*).mp. [mp=protocol supplementary concept, rare disease supplementary concept, title, original title, abstract, name of substance word, subject heading word, unique identifier] (1605262)
41. OR/33-40 (1722995)
42. 9 AND 24 AND 32 AND 41 (374)
