# Supplementary material for: Risks and benefits of stress ulcer prophylaxis in adult neurocritical care patients: a systematic review and meta-analysis of randomized controlled trials
Source: Crit Care. 2015 Nov 17;19:409. doi: 10.1186/s13054-015-1107-2 (PMC4650140; doi:10.1186/s13054-015-1107-2)
Supplement: Additional file 2: — Risk of bias table. (DOCX 109 kb) [file 13054_2015_1107_MOESM2_ESM.docx]

**Risk of bias table**

**Burgess 1995**

| **Bias** | **Authors' judgement** | **Support for judgement** |
| --- | --- | --- |
| Random sequence generation (selection bias) | Low risk | Quote: “each patient was randomly assigned to receive either 6.25 m/hr continuous intravenous ranitidine infusion or a saline placebo infusion in accordance with a computer-generated randomization scheme”. |
| Allocation concealment (selection bias) | Low risk | Quote: “each patient was randomly assigned to receive either 6.25 m/hr continuous intravenous ranitidine infusion or a saline placebo infusion in accordance with a computer-generated randomization scheme”.  Comment: Central allocation |
| Blinding of participants and personnel (performance bias) | Low risk | Quote: “To maintain the integrity of the study blinding and to avoid potential bias, the principal investigator did not have access to the pH data.” |
| Blinding of outcome assessment (detection bias) | Unclear risk | Comment: No information provided. |
| Incomplete outcome data (attrition bias) | High risk | One ranitidine and two placebo-treated patients had pH electrode malfunctions. At least 25% of the pH readings recorded during at least one of the 24-hr time intervals for these three patients were classified as invalid. As a result, all pH readings within these 24-hr time intervals were excluded from the intragastric pH analyses.  Comment: potentially inappropriate application of simple imputation. |
| Selective reporting (reporting bias) | Low risk | The study protocol is available and all of the study’s pre-specified (primary and secondary) outcomes that are of interest in the review have been reported in the pre-specified way. |

**Chan 1995**

| **Bias** | **Authors' judgement** | **Support for judgement** |
| --- | --- | --- |
| Random sequence generation (selection bias) | Low risk | Quote: “all patients were randomized in a standard double-blind manner to receive either ranitidine (50 mg) or placebo medication (normal saline) identical in appearance and volume”. |
| Allocation concealment (selection bias) | Low risk | Quote: “all patients were randomized in a standard double-blind manner to receive either ranitidine (50 mg) or placebo medication (normal saline) identical in appearance and volume”.  Comment: drug containers have identical appearance |
| Blinding of participants and personnel (performance bias) | Low risk | Quote: “all patients were randomized in a standard double-blind manner to receive either ranitidine (50 mg) or placebo medication (normal saline) identical in appearance and volume”. |
| Blinding of outcome assessment (detection bias) | Unclear risk | Comment: No information provided. |
| Incomplete outcome data (attrition bias) | Low risk | Comment: No missing outcome data. |
| Selective reporting (reporting bias) | Unclear risk | Patient outcome: only deaths directly due to gastroduodenal bleeding is reported.  Comment: One outcome of interest in the review are reported incompletely so that they cannot be entered in a meta-analysis; however no reasons for missing data provided, can't be judged as related to true outcome or not. |

**Halloran 1980**

| **Bias** | **Authors' judgement** | **Support for judgement** |
| --- | --- | --- |
| Random sequence generation (selection bias) | Unclear risk | Quote: “Fifty patients with severe head injury were accepted for randomization”.  Comment: Insufficient information about the sequence generation process. |
| Allocation concealment (selection bias) | Low risk | Quote: “Patients were randomly given a coded medication (Smith, Kline, and French Laboratories, Philadelphia, Pennsylvania) in a standard double-blind fashion”. |
| Blinding of participants and personnel (performance bias) | Low risk | Quote: “Patients were randomly given a coded medication in a standard double-blind fashion”.  “No information on gastric secretory data was available to the endoscopist during these studies.” |
| Blinding of outcome assessment (detection bias) | Unclear risk | Comment: Insufficient information provided. |
| Incomplete outcome data (attrition bias) | Unclear risk | Quote: “we did not observe a difference in the incidence of mucosal lesions between the groups in this study, it should be emphasized that the number of patients undergoing endoscopy in each group was small” 14/26 in cimetidine group and 11/24 in placebo group underwent endoscopy.  Comment: Reason for missing outcome data can't be judged as related to true outcome or not. |
| Selective reporting (reporting bias) | Low risk | The study protocol is available and all of the study’s pre-specified (primary and secondary) outcomes that are of interest in the review have been reported in the pre-specified way. |

**Liu 2013**

| **Bias** | **Authors' judgement** | **Support for judgement** |
| --- | --- | --- |
| Random sequence generation (selection bias) | Low risk | Quote: “Patients with negative gastric occult blood testing results at admission were randomized into 3 groups using a computer-generated random numbers table.” |
| Allocation concealment (selection bias) | Low risk | Quote: “Patients with negative gastric occult blood testing results at admission were randomized into 3 groups using a computer-generated random numbers table.”  Comment: Central allocation. |
| Blinding of participants and personnel (performance bias) | High risk | Quote: “This study has a design flaw in that it was not blinded, which allowed for the possibility that the groups were not randomly treated with respect to their overall care.” |
| Blinding of outcome assessment (detection bias) | High risk | Comment: Blinding of outcome assessment, but likely that the blinding could have been broken, and the outcome measurement is likely to be influenced by lack of blinding. |
| Incomplete outcome data (attrition bias) | Unclear risk | Quote: “A total of 184 patients met the inclusion criteria and thus were recruited. Of them, 19 were excluded from data analysis because 11 were lost to follow-up within 30 days of ictus, 5 were not assessable due to missing important data, and 3 did not meet the enrollment criteria. Our results are therefore based on 165 patients who completed the prophylaxis treatment.”  "Data regarding pH levels were available in 144 randomized patients”.  Comment: Missing outcome data balanced in numbers across intervention groups, with similar reasons for missing data across groups; however the missing data can't be judged as related to true outcome or not. |
| Selective reporting (reporting bias) | Low risk | The study protocol is available and all of the study’s pre-specified (primary and secondary) outcomes that are of interest in the review have been reported in the pre-specified way. |

**Metz 1993**

| **Bias** | **Authors' judgement** | **Support for judgement** |
| --- | --- | --- |
| Random sequence generation (selection bias) | Low risk | Quote: “each patient was randomly assigned to receive either 6.25 m/hr continuous intravenous ranitidine infusion or a saline placebo infusion in accordance with a computer-generated randomization scheme”. |
| Allocation concealment (selection bias) | Low risk | Quote: “each patient was randomly assigned to receive either 6.25 m/hr continuous intravenous ranitidine infusion or a saline placebo infusion in accordance with a computer-generated randomization scheme”.  Comment: Central allocation |
| Blinding of participants and personnel (performance bias) | Low risk | Quote: “double-blind”.  Comment: probably done. |
| Blinding of outcome assessment (detection bias) | Unclear risk | Comment: Not sufficient information. |
| Incomplete outcome data (attrition bias) | Low risk | Quote: “No patients were excluded from the study analysis.”  Comment: No missing outcome data. |
| Selective reporting (reporting bias) | Low risk | Quote: “No patients were excluded from the study analysis.”  Comment: The study protocol is available and all of the study’s pre-specified (primary and secondary) outcomes that are of interest in the review have been reported. |

**Misra 2005**

| **Bias** | **Authors' judgement** | **Support for judgement** |
| --- | --- | --- |
| Random sequence generation (selection bias) | Low risk | Quote: “The patients were randomized using computer-generated random table number into 3 groups”. |
| Allocation concealment (selection bias) | Low risk | Quote: “The patients were randomized using computer-generated random table number into 3 groups”.  Comment: Central allocation. |
| Blinding of participants and personnel (performance bias) | High risk | Quote: “The blinding was done by giving placebo injection (saline) or solution (starch).”  Quote: “If the patient developed GH in the placebo group, he was again randomized to one of the treatment options, i.e. ranitidine or sucralfate. If patients on therapy developed GH, he was given a combination of ranitidine and sucralfate.”  Comment: probably not done due to the second randomization after GH. |
| Blinding of outcome assessment (detection bias) | Low risk | Quote: “To prevent bias, randomization was done by one investigator (JK) and evaluation by another (SP) who was unaware about the treatment arm.” |
| Incomplete outcome data (attrition bias) | Low risk | Quote: “One patient each in study and control group was not available for follow up; therefore, they have been excluded from secondary outcome analysis.”  Comment: Missing outcome data balanced in numbers across intervention groups, and are unlikely to be related to true outcome. |
| Selective reporting (reporting bias) | Low risk | The study protocol is available and all of the study’s pre-specified (primary and secondary) outcomes that are of interest in the review have been reported in the pre-specified way. |

**Reusser 1990**

| **Bias** | **Authors' judgement** | **Support for judgement** |
| --- | --- | --- |
| Random sequence generation (selection bias) | Unclear risk | Quote: “Patients were randomized to a treatment or control group within 12 h or ICU admission.”  Comment: Insufficient information about the sequence generation process. |
| Allocation concealment (selection bias) | Unclear risk | Quote: “Patients were randomized to a treatment or control group within 12 h or ICU admission.”  Comment: Insufficient information |
| Blinding of participants and personnel (performance bias) | High risk | Quote: “The need to monitor gastric pH to determine intensification of stress lesion prophylaxis in the treatment group prevented a double-blind study design.” |
| Blinding of outcome assessment (detection bias) | Unclear risk | Comment: Insufficient provided. |
| Incomplete outcome data (attrition bias) | High risk | Quote: “Of 97 eligible patients, 40 (41%) completed the trial. The remainder were excluded for the following reasons: early consent was unobtainable in 12 patients; 14 patients were overlooked by the housestaff; 19 were not endoscoped because the endoscopy team was not available for study purpose; one patient had a duodenal ulcer on initial endoscopy; one did not tolerate repeat endoscopy on day 5; seven were extubated within 48 h; and three died within 48 h due to neurologic deterioration. No excluded patient experienced over GI bleeding.”  Comment: reason for missing outcome data likely to be related to true outcome, with prabable imbalance in numbers and/or reasons for missing data across intervention groups. |
| Selective reporting (reporting bias) | Low risk | The study protocol is available and all of the study’s pre-specified (primary and secondary) outcomes that are of interest in the review have been reported in the pre-specified way. |

**Zhang 2014**

| **Bias** | **Authors' judgement** | **Support for judgement** |
| --- | --- | --- |
| Random sequence generation (selection bias) | High risk | Sequence generated by rules based on date (or day) of admission  Comment: not randomized |
| Allocation concealment (selection bias) | High risk | Allocation based on date (or day) of admission |
| Blinding of participants and personnel (performance bias) | High risk | Comment: No blinding or incomplete blinding, and the outcome is likely to be influenced by lack of blinding. |
| Blinding of outcome assessment (detection bias) | High risk | Comment: No blinding of outcome assessment, and the outcome measurement is likely to be influenced by lack of blinding. |
| Incomplete outcome data (attrition bias) | Low risk | Comment: No missing outcome data. |
| Selective reporting (reporting bias) | Unclear risk | No detailed information on baseline patient characteristics, no comparison of baseline characteristics between treatment and control groups.  Comment: fails to include results that would be expected to have been reported for such a study, however this is not an outcome measure and the influence on results is unclear |
